# Understanding the genetic architecture of the metabolically unhealthy normal weight and metabolically healthy obese phenotypes in a Korean population

**DOI:** 10.1038/s41598-021-81940-y

**Published:** 2021-01-26

**Authors:** Jae-Min Park, Da-Hyun Park, Youhyun Song, Jung Oh Kim, Ja-Eun Choi, Yu-Jin Kwon, Seong-Jin Kim, Ji-Won Lee, Kyung-Won Hong

**Affiliations:** 1 Department of Family Medicine, Gangnam Severance Hospital, Yonsei University College of Medicine, 211 Eonju‐ro, Gangnam-gu, Seoul, 06273 Korea; 2 Department of Medicine, Graduate School of Medicine, Yonsei University, 50-1 Yonsei-ro, Seodaemun-gu, Seoul, 03722 Korea; 3 Theragen Bio Co., Ltd., 145 Gwanggyo-ro, Suwon-si, Gyeonggi-do 16229 Korea; 4 Department of Family Medicine, Yongin Severance Hospital, Yonsei University College of Medicine, 363 Dongbaekjukjeon-daero, Giheung-gu, Yongin-si, Gyeonggi-do 16995 Korea

**Keywords:** Biomarkers, Endocrinology, Medical research

## Abstract

Understanding the mechanisms underlying the metabolically unhealthy normal weight (MUHNW) and metabolically healthy obese (MHO) phenotypes is important for developing strategies to prevent cardiometabolic diseases. Here, we conducted genome-wide association studies (GWASs) to identify the MUHNW and MHO genetic indices. The study dataset comprised genome-wide single-nucleotide polymorphism genotypes and epidemiological data from 49,915 subjects categorised into four phenotypes—metabolically healthy normal weight (MHNW), MUHNW, MHO, and metabolically unhealthy obese (MUHO). We conducted two GWASs using logistic regression analyses and adjustments for confounding variables (model 1: MHNW versus MUHNW and model 2: MHO versus MUHO). *GCKR, ABCB11*, *CDKAL1*, *LPL*, *CDKN2B*, *NT5C2*, *APOA5*, *CETP*, and *APOC1* were associated with metabolically unhealthy phenotypes among normal weight individuals (model 1). *LPL*, *APOA5*, and *CETP* were associated with metabolically unhealthy phenotypes among obese individuals (model 2). The genes common to both models are related to lipid metabolism (*LPL*, *APOA5*, and *CETP*), and those associated with model 1 are related to insulin or glucose metabolism (*GCKR*, *CDKAL1*, and *CDKN2B*). This study reveals the genetic architecture of the MUHNW and MHO phenotypes in a Korean population-based cohort. These findings could help identify individuals at a high metabolic risk in normal weight and obese populations and provide potential novel targets for the management of metabolically unhealthy phenotypes.

## Introduction

Obesity is associated with numerous metabolic disorders, including type 2 diabetes mellitus^1^, dyslipidemia^2^, hypertension^3^, cardiovascular disease^4^, and cancers^5^, which are leading causes of mortality in adults. The ongoing worldwide obesity epidemic constitutes an enormous public health burden^6^. However, not all individuals with obesity have cardiometabolic complications despite excess adiposity; this phenotype is called metabolically healthy obese (MHO). A commonly used definition for MHO requires individuals to be obese and lack metabolic abnormalities^7^. Conversely, individuals with normal weight occasionally exhibit metabolic abnormalities that are usually observed in individuals with obesity, and this phenotype is termed metabolically unhealthy normal weight (MUHNW)^8^. Since there are no universally accepted standard definitions of MHO and MUHNW, the prevalence of these phenotypes heavily depends of the definition that is being used for the characterisation of metabolic health. According to a study conducted using National Health and Nutrition Examination Surveys in the US, 31.7% of obese individuals were metabolically healthy, and 23.5% of normal weight individuals were metabolically unhealthy^9^. In Korea, an estimated 33%–48% of the population with obesity is reported to be MHO, and 12%–21% of individuals with normal weight are reported to be MUHNW^10,11^.

Although the mechanisms that determine why some individuals with obesity remain free from metabolic complications while others with normal weight are susceptible to metabolic complications are not fully understood, previous studies have shown the biological mechanisms possibly associated with the MHO and MUHNW phenotypes, apart from demographic factors (e.g., age, sex, and ethnicity) and environmental factors (e.g., physical activity, smoking, and alcohol intake). These studies indicated that reduced abdominal fat mass and increased gluteofemoral fat mass are associated with the metabolically healthy phenotype, whereas elevated abdominal fat mass and lower gluteofemoral fat mass contribute to the metabolically unhealthy phenotype^12–14^. In addition to fat accumulation and distribution, lipodystrophy, adipogenesis, inflammation, and mitochondrial function are reported to be key contributors to the MHO and MUHNW phenotypes^13–17^.

Over the past decade, genome-wide association studies (GWASs) have been used to identify genetic variants associated with a wide range of diseases and traits. GWASs have identified various genetic loci associated with adiposity, fat distribution, insulin resistance, and metabolic diseases, including hypertension, dyslipidemia, and type 2 diabetes^18–22^. Elucidating such genetic variations can provide insights into the proteins and pathways involved in the development of the MHO and MUHNW phenotypes. Indeed, several GWASs on body fat percentage showed that the genetic variants of certain genes, such as *IRS1*, could be associated with the MUHNW and MHO phenotypes^23–25^. However, few studies have specifically investigated the genetic variants associated with the MUHNW or MHO phenotypes in Asian populations. Here, we conducted GWASs to identify candidate genes harbouring single-nucleotide polymorphisms (SNPs) associated with the MHO and MUHNW phenotypes in a large Korean population-based cohort.

## Results

### Clinical characteristics of the study participants

The clinical characteristics of the participants are described in Table 1. The percentages of individuals with metabolically healthy normal weight (MHNW), MUHNW, MHO, and metabolically unhealthy obese (MUHO) phenotypes among the study population were 47.0% (23 466 individuals), 20.8% (10 358 individuals), 14.0% (7 008 individuals), and 18.2% (9 083 individuals), respectively. The cardiometabolic variables differed significantly among the four groups. We observed a clear elevation in cardiometabolic variables in metabolically unhealthy individuals (MUHNW and MUHO). The prevalence of hypertension and diabetes was also significantly higher among participants with the MUHNW or MUHO phenotypes than among those with the MHNW or MHO phenotypes.

**Table 1:** Clinical characteristics of the study participants categorised into the four obesity phenotypes.

	MHNW (n = 23 466)	MUHNW (n = 10 358)	MHO (n = 7 008)	MUHO (n = 9 083)	*p*-value	*p*-value^1^	*p*-value^2^	*p*-value^3^
Age (years)	51.4 ± 7.0	56 ± 7.6	52.9 ± 7.9	55.3 ± 7.8	< 0.001	< 0.001	< 0.001	< 0.001
Sex					< 0.001	< 0.001	< 0.001	< 0.001
Male (%)	27.8	39.5	38.5	47.3				
Female (%)	72.1	60.5	61.5	52.7				
BMI (kg/m^2^)	22.1 ± 1.8	22.9 ± 1.5	26.8 ± 1.7	27.4 ± 2.1	< 0.001	< 0.001	< 0.001	< 0.001
SBP (mmHg)	116.7 ± 12.9	128.1 ± 14.3	122.2 ± 13.3	131.1 ± 14.2	< 0.001	< 0.001	< 0.001	< 0.001
DBP (mmHg)	72.5 ± 8.9	78.7 ± 9.4	76.0 ± 9.2	80.8 ± 9.5	< 0.001	< 0.001	< 0.001	< 0.001
FPG (mg/dL)	89.0 ± 11.1	102.6 ± 25.4	90.9 ± 11.7	105.1 ± 25.7	< 0.001	< 0.001	< 0.001	< 0.001
Total cholesterol (mg/dL)	195.8 ± 33.3	200.3 ± 38.1	201.6 ± 34	201.3 ± 37.5	< 0.001	< 0.001	0.020	< 0.001
TG (mg/dL)	89.9 ± 43.6	167.7 ± 104.7	105.1 ± 50.1	186.4 ± 113	< 0.001	< 0.001	< 0.001	< 0.001
HDL-C (mg/dL)	59.1 ± 12.7	48.2 ± 12.0	55 ± 11.1	46.1 ± 10.6	< 0.001	< 0.001	< 0.001	< 0.001
Hypertension (%)	1 516 (6.4)	3 313 (32.0)	911 (13.0)	3 765 (41.4)	< 0.001	< 0.001	< 0.001	< 0.001
Diabetes (%)	351 (1.5)	1 430 (13.8)	110 (1.6)	1 320 (14.5)	< 0.001	< 0.001	< 0.001	< 0.001
Regular exerciser (%)	9 071 (39.4)	4 120 (40.6)	2 726 (39.8)	3 453 (38.7)	0.401	0.039	0.179	0.265
Smoker (%)					< 0.001	< 0.001	< 0.001	< 0.001
Current smoker (%)	2 215 (9.4)	1 415 (13.7)	776 (11.1)	1 386 (15.3)				
Former smoker (%)	2 749 (11.7)	1 791 (17.3)	1 244 (17.8)	2 011 (22.1)				
Drinker (%)	3 777 (14.4)	1 928 (18.6)	1 341 (19.1)	2 017 (22.2)	< 0.001	< 0.001	< 0.001	0.388

Data are presented as the mean ± standard deviation or percentage. We obtained *p*-values by one-way analysis of variance or independent two-sample *t*-tests for continuous variables or by χ^2^ tests for categorical variables.

*p*-values represent the difference in each variable among the MHNW, MUHNW, MHO and MUHO phenotypes.

MHNW, metabolically healthy normal weight; MUHNW, metabolically unhealthy normal weight; MHO, metabolically healthy obese; MUHO, metabolically unhealthy obese; BMI, body mass index; SBP, systolic blood pressure; DBP, diastolic blood pressure; FPG, fasting plasma glucose; TG, triglyceride; HDL-C, high-density lipoprotein cholesterol.

*p*-values^1^ represent the difference in each variable between the MHNW and MUHNW phenotypes.

*p*-values^2^ represent the difference in each variable between the MHO and MUHO phenotypes.

*p*-values^3^ represent the difference in each variable between the MUHNW and MHO phenotypes.

### Genetic regions associated with metabolically unhealthy phenotypes in model 1 (MHNW versus MUHNW) and model 2 (MHO versus MUHO)

We conducted a GWAS for model 1 to identify the genetic factors associated with the metabolically unhealthy phenotype among the normal weight groups and for model 2 to identify the genetic factors associated with the metabolically unhealthy phenotype among the obese groups.

The lead SNPs and clusters of SNPs in the regions associated with the metabolically unhealthy phenotype in models 1 and 2 are shown in Tables 2-1 and 2-2, respectively. Odds ratios (ORs) and 95% confidence intervals (CIs) were calculated using logistic regression analysis after adjusting for age, sex, exercise status, smoking status, alcohol intake, body mass index (BMI), and principle component (PC) 1 and PC2. SNPs in *LPL*, *APOA5*, and *CETP* exhibited significant association with the risk of metabolically unhealthy phenotypes in both normal weight and obese individuals (in models 1 and 2). SNPs in *GCKR*, *ABCB11*, *CDKAL1*, *CDKN2B*, *NT5C2*, and *APOC1* were significantly associated with the risk of metabolically unhealthy phenotypes only in normal weight individuals (in model 1 only).

**Table 2:** Representative SNPs identified by GWAS among the significant loci for model 1 (MHNW versus MUHNW) and model 2 (MHO versus MUHO).

Gene	Region	Chr	Position (bp)	SNP	SNP cluster	Minor allele	MAF	OR (95% CIs)	*p*-value
2–1. *MHNW (control) versus MUHNW (case)*									
*GCKR*	Intron variant	2	27 734 972	rs6547692	rs780096, rs1260326	A	0.46	0.90 (0.87–0.94)	1.27E-08
ABCB11	Intron variant	2	169 803 568	rs16856261	rs3755157, rs58512362	T	0.37	1.12 (1.08–1.16)	2.07E-09
*CDKAL1*	Intron variant	6	20 693 697	rs138420022	rs34499031, rs35261542	A	0.47	1.15 (1.11–1.19)	1.09E-14
*LPL*	Downstream gene variant	8	19 865 455	rs77237194	rs10096633, rs17482753	T	0.12	0.83 (0.78–0.87)	2.56E-12
*CDKN2B*	Downstream gene variant	9	22 132 729	rs10965247	rs10811660, rs10965246	G	0.44	0.89 (0.86–0.92)	1.27E-10
*NT5C2*	Downstream gene variant	10	104 960 464	rs113278154	rs79237883, rs34747231	T	0.27	0.90 (0.86–0.92)	4.37E-08
*APOA5*	Upstream gene variant	11	116 662 579	rs651821	rs662799, rs2075291	C	0.30	1.47 (1.42–1.53)	1.68E-90
*CETP*	Intron variant	16	57 002 663	rs9926440	rs17231506, rs821840	C	0.31	1.16 (1.11–1.20)	2.95E-14
*APOC1*	Intron variant	19	45 420 082	rs73052335	rs111789331, rs66626994	C	0.10	1.24 (1.17–1.32)	1.24E-13
2–2. *MHO (control) versus MUHO (case)*									
*LPL*	Downstream gene variant	8	19 827 848	rs10105606	rs1441766, rs4464984	A	0.12	0.83 (0.78–0.87)	1.68E-11
*APOA5*	Upstream gene variant	11	116 662 579	rs651821	rs662799	C	0.30	1.43 (1.36–1.51)	8.55E-44
*CETP*	Upstream gene variant	16	56 993 886	rs821840	rs36229491, rs17231506	G	0.17	0.83 (0.78–0.88)	2.02E-10

Logistic regression models were adjusted for age, sex, exercise status, smoking status, alcohol intake, body mass index, and PC1 and PC2.

GWAS, genome-wide association study; MHNW, metabolically healthy normal weight; MUHNW, metabolically unhealthy normal weight; MHO, metabolically healthy obese; MUHO, metabolically unhealthy obese; Chr, chromosome; SNP, single-nucleotide polymorphism; MAF, minor allele frequency; OR, odds radio; CI, confidence interval.

**Figure 1:**
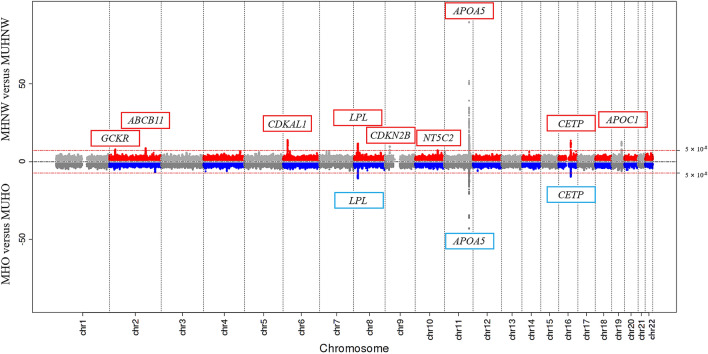
Miami plot of the GWASs for model 1 (MHNW versus MUHNW) and model 2 (MHO versus MUHO). SNP locations are plotted on the x-axis according to their chromosomal position. The −log_10_(*p*-values) derived from the logistic regression analysis are plotted on the y-axis. The *p*-values were adjusted for age, sex, exercise status, smoking status, alcohol intake, body mass index, and PC1 and PC2. The horizontal red line indicates the formal threshold for genome-wide significance at *p* = 5.00 × 10^−8^. GWASs, genome-wide association studies; MHNW, metabolically healthy normal weight; MUHNW, metabolically unhealthy normal weight; MHO, metabolically healthy obese; MUHO, metabolically unhealthy obese; PC, principle component; SNP, single-nucleotide polymorphism. The figure was generated using EasyStrata version 8.6 (http://www.genepi-regensburg.de/easystrata).

The results of the model 1 and model 2 GWASs are illustrated in a Miami plot (Fig. 1), a format recently developed at Michigan University to describe two comparable GWAS results. We observed genome-wide significant association clusters of *GCKR*, *ABCB11*, *CDKAL1*, *LPL*, *CDKN2B*, *NT5C2*, *APOA5*, *CETP*, and *APOC1* in model 1 (upper plot) and *LPL*, *APOA5*, and *CETP* in model 2 (lower plot).

## Discussion

Our study aimed to identify the genetic variations that differentiated the MHO phenotype from the MUHNW phenotype. Determining the genetic characteristics associated with the MHO and MUHNW phenotypes will enable us to pinpoint the biological mechanisms driving these two paradoxical conditions and develop approaches to prevent cardiometabolic diseases. Over the past decade, several genomic studies have identified numerous genetic variants associated with adiposity in the context of a favourable cardiometabolic profile; many of these loci are located in or near genes involved in adipogenesis, fat distribution, and insulin signalling^18,19^. However, few studies have fully utilised genome-wide genetic variants to characterise the MHO and MUHNW phenotypes. Furthermore, the majority of genetic studies have analysed populations of European ancestry, and limited data are available from Asian populations.

We found that *LPL*, *APOA5*, and *CETP* were associated with metabolically unhealthy phenotypes among both normal weight and obese individuals. These three genes are related to lipid metabolism. *LPL*, located on 8p21.3, encodes a key lipolysis regulator. In addition, LPL may link insulin resistance to atherosclerosis because it controls the delivery of free fatty acids to muscles, adipose tissues, and vascular wall macrophages^26^. *APOA5*, a member of the *APOA4*/*APOC3*/*APOA1* gene cluster, is located on chromosome 11q23.3 and plays important roles in lipid metabolism, particularly for triglycerides (TGs) and TG-rich lipoproteins. There is considerable evidence supporting the association between *APOA5* SNPs, such as rs662799 and rs651821, and an increased risk of obesity and metabolic syndrome^27^. Consistent with previous results, we found that rs662799 and rs651821 in *APOA5* were associated with metabolically unhealthy phenotypes. *CETP,* located on chromosome 16q13, encodes the CETP protein that shuttles TGs and cholesteryl ester between lipoproteins^28^. The expression of *CETP* genetic variants has been associated with variations in high-density lipoprotein cholesterol (HDL-C) levels in various ethnic groups^29^. Our results support the link between certain genetic variants and metabolically unhealthy phenotypes.

We also found distinct genetic variants that were associated with metabolically unhealthy phenotypes among normal weight and obese individuals. *GCKR*, *ABCB11*, *CDKAL1*, *CDKN2B*, *NT5C2*, and *APOC1* were associated with metabolically unhealthy phenotypes in individuals with normal weight but not in those with obesity. Of these, *GCKR*, *CDKAL1*, and *CDKN2B* are related to insulin or glucose metabolism. *GCKR*, which maps to chromosome 2p23.3, encodes a protein involved in the regulation of glucokinase activity; the protein modulates glucose balance and glucose-stimulated insulin secretion^30^. Some variants of *GCKR* are associated with insulin, fasting glucose, and TG levels and with susceptibility to type 2 diabetes mellitus^31,32^. Genetic variants of *CDKAL1*, which maps to chromosome 6p22.3, are strongly linked to an increased risk of developing type 2 diabetes and obesity^33^. *CDKAL1* is associated with proinsulin conversion and insulin response upon glucose stimulation^34^ and is necessary for normal mitochondrial morphology and adipose tissue function^33^. In recent studies, the *CDKAL1* rs7754840 variant was shown to be associated with increased waist circumference and waist-to-hip ratio in Chinese Han patients^35^, and the *CDKAL1* rs2206734 polymorphism was shown to be an independent predictor of the MUHNW phenotype in Chinese children^36^. Similarly, our study suggested that *CDKAL1* SNPs are important predictors of the MUHNW phenotype. *CDKN2B*, which is located in chromosome 9p21, plays a role in the deterioration of insulin secretion by participating in the regulation of pancreatic β-cell proliferation and function^37,38^. Studies across varied ethnicities and geographical locations showed that polymorphisms at the *CDKN2A* locus are related to type 2 diabetes mellitus development^39–41^. *ACBC11* located in chromosome 2q24 encodes an ATB-binding cassette transporter. Previous studies have shown that variations in *ABCB11* are associated with increased fasting glucose levels^42–44^. *NT5C2* encodes a hydrolase that plays a key role in cellular purine metabolism and uric acid regulation^45,46^. SNPs in *NT5C2* have been associated with hypertension^47–49^. *APOC1* encodes a member of the apolipoprotein C1 family that plays an important role in HDL-C and very low density lipoprotein metabolism. Previous studies have indicated that some variants of *APOC1* are associated with metabolic abnormalities^50,51^. Our findings suggested that strategies for protecting against complications related to metabolically unhealthy phenotypes might differ for individuals with normal weight and those with obesity. Further studies to identify the potential interactions of these candidate genes using biological and mechanical analyses are required.

Previous GWASs have identified various genetic variants associated with diverse metabolic diseases such as metabolic syndrome^52,53^, dyslipidemia^54,55^, and obesity^56,57^. However, only a limited number of studies have specifically revealed the genetic loci associated with the MUHNW and MHO phenotypes. We performed GWASs for the metabolically unhealthy phenotypes divided into the normal weight and obesity groups to identify the genes and SNPs associated with the MUHNW and MHO phenotypes. The present findings contribute to our understanding of the genetic architecture of the MUHNW and MHO phenotypes.

There are several limitations to consider in the interpretation of our results. The findings were not replicated. Furthermore, since the current study was performed in a Korean population, the findings may not apply to non-Asian populations. Replication studies and studies in other populations are necessary to confirm our findings and determine their applicability to ethnically diverse groups. In addition, there are no universally accepted standard definitions of MHO and MUHNW; we employed definitions that have been widely used in previous studies^58,59^. Despite these potential limitations, we believe our GWASs provide valuable data on the genetic characteristics associated with the MUHNW and MHO phenotypes in a large population-based cohort.

In summary, our GWASs provide several insights into the genetic architecture of metabolically unhealthy individuals. We found that *LPL*, *APOA5*, and *CETP* were associated with the metabolically unhealthy phenotypes in individuals with normal weight or obesity. *GCKR*, *ABCB11*, *CDKAL1*, *CDKN2B*, *NT5C2*, and *APOC1* were associated with metabolically unhealthy in individuals with normal weight but not in those with obesity. Our study provides an understanding of the genetic architecture of the MUHNW and MHO phenotypes in a Korean population-based cohort. Although this study remains to be validated in a larger cohort and followed up with investigation on the pathophysiological pathways involved, our findings could help identify metabolically high-risk individuals in normal weight and obese populations and provide potential novel targets for the management of metabolically unhealthy phenotypes.

## Methods

### Study overview and study participants

The current study used Korean Genome and Epidemiology Study (KoGES) Health Examination data. The cohort consisted of male and female community dwellers recruited from the national health examinee registry (aged 40–79 years at baseline). Eligible participants were asked to volunteer via on-site invitation, letters, telephone calls, media campaign, or community conferences. The responders were invited to visit the survey sites, including medical schools, hospitals, and health institutions, for an interview in which they answered a questionnaire administered by trained staff and underwent physical examination. We collected information on their past medical history, smoking history, alcohol consumption, and physical activity during a health interview. We defined a regular exerciser as an individual who participated in vigorous physical activity more than three times per week. Current smokers were individuals who smoked ≥ 100 cigarettes in their lifetime and were smoker at the time of the study, and former smokers were individuals who smoked ≥ 100 cigarettes in their lifetime but were non-smokers at the time of the study. A drinker was defined as an individual consuming alcoholic beverages at least twice a week. The KoGES participants were all of Korean ethnicity. The detailed history and profile of KoGES were previously published^60^.

In total, 58 701 participants, for whom genome-wide SNP genotype data were available, were included in the KoGES Health Examination dataset. Of these, we excluded the participants who had a history of cancer, thyroid disease, stroke, and/or myocardial infarction (n = 6 965). Participants aged 75 years or older (n = 23) and those with missing data for BMI, blood pressure, fasting plasma glucose, TG, and/or HDL-C levels, and/or exercise/smoking/alcohol were excluded (n = 1 798). After these exclusions, 49 915 participants were included in the final analysis. Written informed consent was obtained from all participants. This research project was approved by the Institutional Review Board of Theragen Etex (approval number: 700062-20190819-GP-006-02). In addition, the study complied with the ethical principles of the Declaration of Helsinki.

### Measurement of anthropometric and laboratory data

Weight and height were measured to the nearest 0.1 kg and 0.1 cm, respectively, with participants wearing light indoor clothing and no shoes. We calculated the BMI as body weight (kg) divided by the square of height (m^2^). Systolic and diastolic blood pressure (SBP and DBP, respectively) were measured twice with a standardised mercury sphygmomanometer (Baumanometer Standby; W.A. Baum, New York, NY, USA). Blood samples were obtained in the morning after overnight fasting. We measured fasting plasma glucose, total cholesterol, TG, and HDL-C levels with an automatic analyser (ADIVA 1650; Siemens, Tarrytown, NY, USA).

### Definition of study phenotypes

We defined a participant with obesity as an individual with BMI ≥ 25 kg/m^2^ based on the Asia–Pacific regional guidelines of the World Health Organization and International Obesity Task Force^61^. Normal weight was defined as BMI < 25 kg/m^2^. A metabolically healthy individual was a participant with less than two of the following four metabolic traits: elevated blood pressure (SBP/DBP ≥ 130/85 mm Hg or taking antihypertensive medication); impaired fasting plasma glucose (≥ 100 mg/dL), diagnosis of diabetes mellitus, and/or prescription for antidiabetic medication; high plasma TG (≥ 150 mg/dL); and low HDL-C (< 40 mg/dL in men or < 50 mg/dL in women). According to these criteria, study participants were categorised into one of four groups: (1) MHNW: BMI < 25 kg/m^2^ and less than two metabolic risk factors; (2) MUHNW: BMI < 25 kg/m^2^ and at least two metabolic risk factors; (3) MHO: BMI ≥ 25 kg/m^2^ and less than two metabolic risk factors; (4) MUHO: BMI ≥ 25 kg/m^2^ and at least two metabolic risk factors.

### Genotyping and quality-control

The genotype data were graciously provided by the Centre for Genome Science, Korea National Institute of Health and were produced using a Korea Biobank Array (Affymetrix, Santa Clara, CA, USA)^62^. The experimental results of the Korea Biobank Array were filtered using the following quality-control criteria: call rate, > 97%; minor allele frequency, > 1%; Hardy–Weinberg equilibrium, *p* < 1 × 10^−5^. After quality-control filtering, the experimental phenotypes were used to analyse the genotype datasets from the 1 000 Genome Phase 1 and 2 Asian panel. The GWASs identified 7 975 321 SNPs on chromosomes 1 to 22.

### Statistical analysis

We compared the clinical characteristics of the study participants with different phenotypes using one-way analysis of variance for continuous variables and χ^2^ tests for categorical variables.

This study included two GWASs. The first GWAS was conducted on metabolically unhealthy individuals from the normal weight groups (model 1: MHNW [control] and MUHNW [case]). The second GWAS was conducted on metabolically unhealthy individuals from the obesity groups (model 2: MHO [control] and MUHO [case]).

We performed PC analysis to reduce the bias of the genomic data according to regions where the samples were collected. Based on this analysis, we obtained PC1 and PC2, which were used as covariates for statistical analysis. All GWASs were conducted using logistic regression analysis after adjusting for age, sex, exercise status, smoking status, alcohol intake, BMI, and PC1 and PC2 as covariates, using PLINK version 1.90. We calculated the ORs and 95% CIs for the GWASs. The SNPs listed in the tables are representative SNPs of approximately 50 kbp with significant *p*-values (*p* < 5.00 × 10^–8^). Significant associations were defined by genome-wide significance level *p*-values < 5.00 × 10^–8^.
